# Vitamin D and COVID-19 severity and related mortality: a prospective study in Italy

**DOI:** 10.1186/s12879-021-06281-7

**Published:** 2021-06-14

**Authors:** Irene Campi, Luigi Gennari, Daniela Merlotti, Christian Mingiano, Alessandro Frosali, Luca Giovanelli, Camilla Torlasco, Martino F. Pengo, Francesca Heilbron, Davide Soranna, Antonella Zambon, Marta Di Stefano, Carmen Aresta, Marco Bonomi, Biagio Cangiano, Vittoria Favero, Letizia Fatti, Giovanni Battista Perego, Iacopo Chiodini, Gianfranco Parati, Luca Persani

**Affiliations:** 1 Department of Endocrine and Metabolic Diseases & Lab of Endocrine and Metabolic Research, IRCCS, Istituto Auxologico Italiano, Via Magnasco 2, 20149 Milan, Italy; 2 Department of Pathophysiology and Transplantation, University of Milan, Milan, Italy; 3 Department of Medicine, Surgery and Neurosciences, University of Siena, Siena, Italy; 4 Department of Clinical Sciences and Community Health, University of Milan, Milan, Italy; 5 Department of Cardiovascular, Neural and Metabolic Sciences, IRCCS, Istituto Auxologico Italiano, Milan, Italy; 6 Department of Medicine and Surgery, University of Milano-Bicocca, 20100 Milan, Italy; 7 Biostatistic Unit, IRCCS Istituto Auxologico Italiano, Milan, Italy; 8 Department of Statistics and Quantitative Methods, Università di Milano-Bicocca, Milan, Italy

**Keywords:** Vitamin D, COVID-19, Mortality, Interleukin-6

## Abstract

**Background:**

Vitamin D deficiency has been suggested to favor a poorer outcome of Coronavirus disease-19 (COVID-19). We aimed to assess if 25-hydroxyvitamin-D (25OHD) levels are associated with interleukin 6 (IL-6) levels and with disease severity and mortality in COVID-19.

**Methods:**

We prospectively studied 103 in-patients admitted to a Northern-Italian hospital (age 66.1 ± 14.1 years, 70 males) for severely-symptomatic COVID-19. Fifty-two subjects with SARS-CoV-2 infection but mild COVID-19 symptoms (mildly-symptomatic COVID-19 patients) and 206 subjects without SARS-CoV-2 infection were controls. We measured 25OHD and IL-6 levels at admission and focused on respiratory outcome during hospitalization.

**Results:**

Severely-symptomatic COVID-19 patients had lower 25OHD levels (18.2 ± 11.4 ng/mL) than mildly-symptomatic COVID-19 patients and non-SARS-CoV-2-infected controls (30.3 ± 8.5 ng/mL and 25.4 ± 9.4 ng/mL, respectively, *p* < 0.0001 for both comparisons). 25OHD and IL-6 levels were respectively lower and higher in severely-symptomatic COVID-19 patients admitted to intensive care Unit [(ICU), 14.4 ± 8.6 ng/mL and 43.0 (19.0–56.0) pg/mL, respectively], than in those not requiring ICU admission [22.4 ± 1.4 ng/mL, *p* = 0.0001 and 16.0 (8.0–32.0) pg/mL, *p* = 0.0002, respectively]. Similar differences were found when comparing COVID-19 patients who died in hospital [13.2 ± 6.4 ng/mL and 45.0 (28.0–99.0) pg/mL] with survivors [19.3 ± 12.0 ng/mL, *p* = 0.035 and 21.0 (10.5–45.9) pg/mL, *p* = 0.018, respectively). 25OHD levels inversely correlated with: i) IL-6 levels (ρ − 0.284, *p* = 0.004); ii) the subsequent need of the ICU admission [relative risk, RR 0.99, 95% confidence interval (95%CI) 0.98–1.00, *p* = 0.011] regardless of age, gender, presence of at least 1 comorbidity among obesity, diabetes, arterial hypertension, creatinine, IL-6 and lactate dehydrogenase levels, neutrophil cells, lymphocytes and platelets count; iii) mortality (RR 0.97, 95%CI, 0.95–0.99, p = 0.011) regardless of age, gender, presence of diabetes, IL-6 and C-reactive protein and lactate dehydrogenase levels, neutrophil cells, lymphocytes and platelets count.

**Conclusion:**

In our COVID-19 patients, low 25OHD levels were inversely correlated with high IL-6 levels and were independent predictors of COVID-19 severity and mortality.

## Background

The consequences of infection with SARS-CoV-2 broadly varies from benign to fatal. While many infected individuals remain asymptomatic or experience only mild symptoms (e.g. fever, dyspnea, cough, myalgia, fatigue or less frequently diarrhea), others develop moderate to severe coronavirus disease 2019 (COVID-19), mainly characterized by interstitial pneumonia, frequently progressing to acute respiratory distress syndrome (ARDS) and death from respiratory failure or other complications. Indeed, the occurrence of pneumonia requiring respiratory support with continuous positive airway pressure treatment or non-invasive ventilation, seems a critical event characterizing asymptomatic or mild cases, from those with more severe disease, which may progress towards severe acute respiratory failure (in need of invasive mechanical ventilation), with up to 95% prevalence of respiratory distress described in patients who died for the disease [1].

It is believed that SARS-CoV-2 outcomes may be determined by the extent of the host immune system imbalance [2–4]. While the primary immune response exerts a positive effect against infection, facilitating viral clearance, the secondary immune response, at least in a subset of subjects, may be exaggerated and challenge tissue integrity, thus leading to multiple organ failure, ARDS and death [2]. Consistent with this hypothesis, together with older age and the presence of major comorbidities (e.g. diabetes, obesity, hypertension and cardiovascular diseases), predictors of COVID-19 fatality mainly include elevated cytokine levels or other inflammatory markers [5] suggesting that mortality might be somewhat related to virally driven hyper-inflammation, known as “cytokine storm” [3, 4].

In this COVID-19 emergency, given the complex pathophysiology of this disease, information media have sometimes conveyed inaccurate or biased information based on anecdotal or insufficiently supported data. One of these debated issues is the role of vitamin D in modulating the severity of COVID-19, as this vitamin exerts well known immuno-modulatory functions spanning from the innate to the adaptive arms of the immune system and including the downregulation of pro-inflammatory cytokines [6], such as interleukin-6 (IL-6). A wealth of clinical and preclinical observations suggested that insufficient vitamin D levels may favor viral infections, particularly in the respiratory tract, as well as autoimmune disorders [6, 7]. Indeed, the clinical connection between vitamin D and infections stemmed initially from the recognition that sunlight, likewise the use of cod liver oil, were beneficial for patients suffering from tubercolosis. Then, other studies demonstrated that vitamin D supplementation protects against respiratory infections [8, 9] and that there is an association between vitamin D deficiency [i.e. serum 25-hydroxyvitamin D (25OHD) levels below 20 ng/mL] and increased risk of progression and death from viral infections such as HIV, due to persistent immune activation, greater inflammation and activated monocyte phenotypes [9]. This relationship seems independent from comorbid conditions, that, likewise in COVID-19, represent well known determinants of HIV severity and mortality [10]. In this respect, a recent systematic literature revision from the UK Government Scientific Advisory Committee on Nutrition (SACN) suggested that, albeit stronger evidence should be required, there may be some benefit from daily, low-dose vitamin D supplementation (between 10 and 25 μg/day; 400 to 1000 IU/day) in reducing risk of acute respiratory tract infections [11].

Based on all the above premises, it is not surprising that information media, physicians and even health authorities had been debating about the possibility that vitamin D replacement might represent an useful intervention to strengthen the immune system and help in the fight against COVID-19 [12]. Such a possibility is further supported by recent reviews of epidemiology data, suggesting a likely relationship between COVID-19 severity and the prevalence of vitamin D deficiency in a population [13–15], as well as from small reports in patients with or without COVID-19 [16–22]. However, it still remains to be demonstrated, on a prospective basis and taking into account possible confounding factors (e.g. age, gender and major comorbidities), whether vitamin D deficiency or low 25OHD levels, as assessed at the time of diagnosis, are associated with the severity of COVID-19, a demonstration that would be required to further support the planning of clinical trials aimed at assessing the efficacy of vitamin D administration in patients infected with SARS-CoV-2.

Therefore, we aimed to prospectively investigate, in a well characterized cohort of consecutive COVID-19 patients admitted to our COVID-19 hospital, the association between 25OHD levels at hospital admission and COVID-19 severity and related mortality during the course of hospitalization.

## Methods

### Subjects

We recruited a cohort of 103 consecutive, white Caucasian patients affected with severely-symptomatic COVID-19 (severely-symptomatic COVID-19 patients), admitted to the COVID-19 Units of San Luca Hospital, Istituto Auxologico Italiano in Milan, Italy, due to respiratory insufficiency between March 9th and April 30th 2020. The criteria for inclusion in the severely-symptomatic COVID-19 patients group were acute respiratory failure (namely, spontaneous oxygen saturation ≤ 93% and/or PaO2/FiO2 ratio < 300 mmHg) requiring invasive or non-invasive ventilation, with or without the presence of fever (> 37.5 °C) and other organ dysfunction.

These patients were matched by age, weight and gender with a 206 (2:1 ratio) non SARS-CoV-2 infected controls derived from a reference population of 3174 consecutive subjects (defined from now on as controls), who underwent 25OHD measurement in Siena (Italy) between January 01 and March 31, 2020, during a medical check-up, regardless of hospitalization status (with up to 65% of assessments deriving from non-hospitalized subjects). These individuals were included in the control group if they had no epidemiological and clinical evidence of SARS-CoV-2 infection at the time of 25OHD measurement.

Moreover, during March–April 2020 period, a cohort of 52 subjects was recruited as a reference group with milder disease (so called “mildly-symptomatic” COVID-19 patients). The inclusion criteria for the “mildly-symptomatic” COVID-19 cases were the laboratory evidence of SARS-Cov-2 infection with only mild symptoms of COVID-19 (e.g. fever, dry cough, tiredness, loss of taste or smell) but without respiratory failure. These mildly-symptomatic COVID-19 subjects were consecutively recruited from either the Istituto Auxologico Italiano Nursing Home (*n* = 20, age 86.5 ± 6.8 years) or among the Istituto Auxologico Italiano employees who were screened for SARS-Cov-2 infection (*n* = 32, age 49.6 ± 10.2 years).

**Fig. 1:**
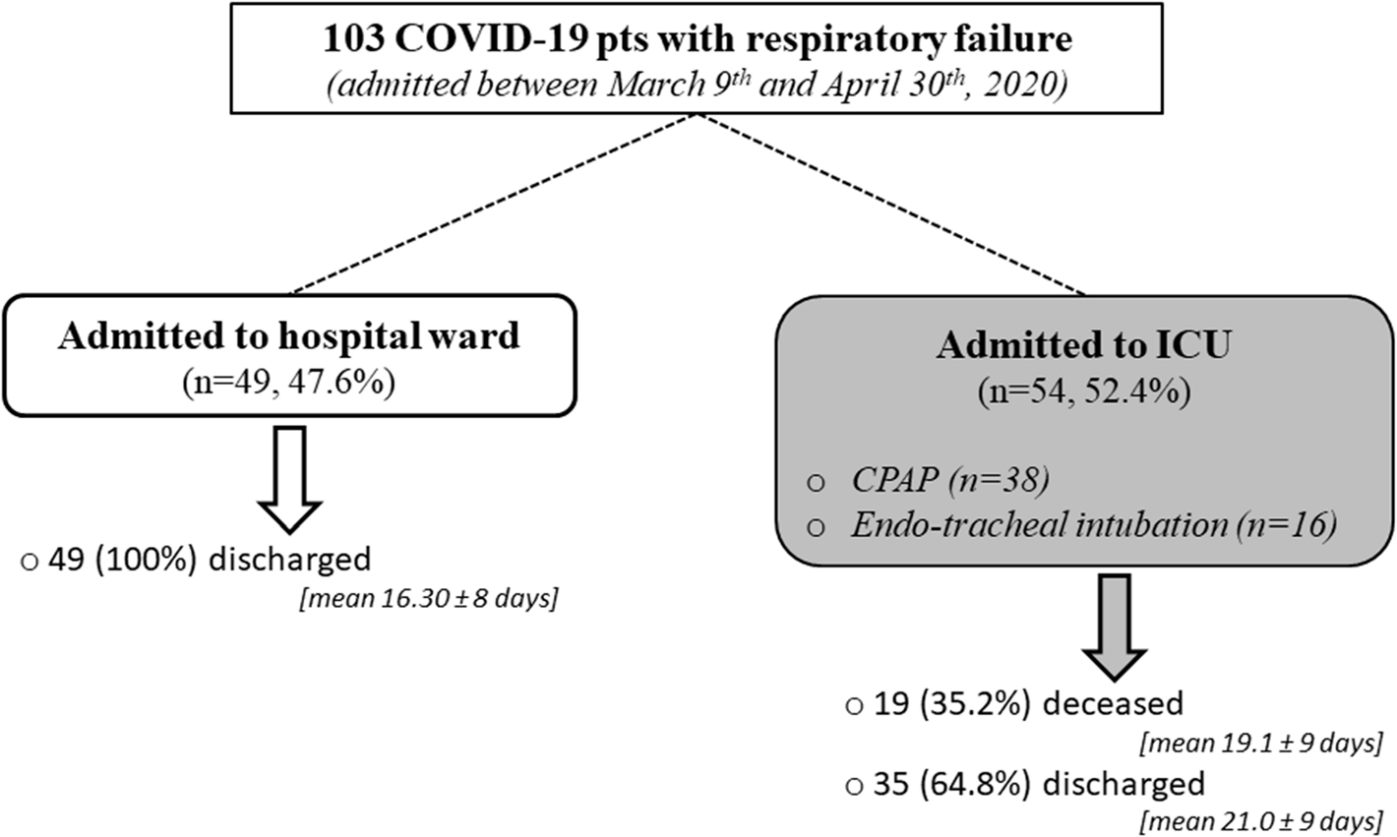
Clinical Course of Symptomatic COVID-19 Cases

Overall, our study included 361 subjects (52 mildly-symptomatic COVID-19 patients, 103 severely-symptomatic COVID-19 patients and 206 non SARS-CoV-2 infected controls) with 25OHD measurement. For the severely-symptomatic COVID-19 patients, data about disease progression and in-hospital mortality were recorded. During the course of the disease in-hospital, 54 severely-symptomatic COVID-19 patients needed to be admitted to the intensive care unit (ICU) with ventilator support through either CPAP (*n* = 38) and/or endo-tracheal intubation (*n* = 16), while the remaining patients (*n* = 49) remained in a ward (Fig. 1). The indication for invasive or non-invasive mechanical ventilation was mainly based on the Brescia-COVID Respiratory Severity Scale (BCRSS)/Algorithm [23].

Among mildly-symptomatic COVID-19 patients and severely-symptomatic COVID-19 patients, 8 (15.3%) and 13 (12.6%, p = n.s.) subjects, respectively, were affected by chronic obstructive pulmonary disease, and 41 (78.8%) and 31 (30.1%, *p* < 0.005), respectively, were taking cholecalciferol supplements. No differences in gender distribution, age, BMI and comorbidities were found between patients supplemented with cholecalciferol and those not taking cholecalciferol supplements in both mildly-symptomatic COVID-19 and severely-symptomatic COVID-19 groups (data not shown). Among severely-symptomatic COVID-19 patients 8, 23 and 53 subjects were treated with tocilizumab, dexamethasone and chloroquine, respectively, during hospitalization. Five patients (3 females and 2 males) had serum creatinine levels above the upper normal limits (106.1 μmol/L for men and 88.4 μmol/L for women), of which 2 were above 176.8 μmol/L. The diabetes control was inadequate (i.e. glycated hemoglobin above 58 mmol/mol) in 4 and 2 subjects with severely-symptomatic or mildly-symptomatic COVID-19 (p = n.s.), respectively.

### Methods

In all patients the diagnosis of SARS-Cov-2 infection was defined as a positive result from naso-pharyngeal swab associated with reverse transcription-polymerase chain reaction analysis (GeneXpert, Cepheid, Sunnyvale, USA). In all subjects, circulating 25OHD levels (25OHD, LIASON 25-OH Vitamin D TOTAL Assay; Diasorin Diagnostics) were measured. For each patient we defined the presence of vitamin D deficiency in case of 25OHD ≤20 ng/mL. The measurement of 25OHD levels was performed at hospital admission in severely-symptomatic COVID-19 patients, as well as at the time of SARS-CoV-2 infection diagnosis in mildly-symptomatic COVID-19 cases. In all subjects the body mass index was directly calculated or derived from medical reports, and information concerning demographics, medical history and the presence of common comorbidities (i.e. hypertension, type 2 diabetes, obesity) was recorded within the first 24 h following SARS-Cov-2 infection diagnosis. In all hospitalized COVID-19 patients, at admission, we also assessed serum IL-6 (electrochemiluminescence assay, Roche Diagnostics, normal values < 7 pg/mL), C-reacting protein levels (by immune-turbidimetric method), lactate dehydrogenase, calcium, creatinine, D-dimer, and blood count by automatic auto-analyzer.

### Statistical analysis

An unambiguous and progressive alphanumeric code was assigned to all patients and controls, in order to obtain an anonymously recorded database. Continuous variables are shown as mean and standard deviation (SD) for data with normal distribution (evaluated with Kolmogorov-Smirnov test) and median and interquartile range (IQR) in case of no normal data. Categorical data are reported as frequencies and proportions. Comparisons of continuous variables among more than two groups were carried out by means of ANOVA model (or Kruskal-wallis test in case of no normal data), while T-test (or Wilcoxon test) was used to compare continuous variables between two groups. Chi-square and Fisher test were used to compare categorical variables among groups. To compare the mean level of 25OHD among groups adjusting for other covariates we applied an ANOVA model with Tukey method to control the inflation of type I error due to head to head comparisons. Correlation between two continuous variables were tested by Pearson coefficient (or Spearman in case of no normal distribution). Two multivariable Poisson regression models with robust variance were implemented to estimate the association between 25OHD and the need of admission to ICU or death [24]. These association estimates were reported as Relative Risk (RR) and its relative 95% confidence interval (95%CI). These associations were adjusted for possible confounding factors (age and gender) and/or for the variables (e.g. biomarkers and major comorbidities) that were found to be statistically different (*p*-value < 0.05) between patients who needed or not the admission to ICU or who died or survived, respectively. All analyses were performed by two statisticians (DS and AZ) using the Statistical Analysis System Software (version 9.4; SAS Institute, Cary, NC). Statistical significance was set at the 0.05 level. All *P* values were 2-sided.

A sample size of 95 patients was estimated in order to identify as significant a 3% reduction in the risk of death assuming I type error of 5%, a power of 80% and a proportion of deaths of 15% (taking into account a 13–18% of death rates for Covid-19, as observed in Lombardia region during the first pandemic flow). This sample size was calculated considering a logistic model with one continuous predictor and it was based on the likelihood ratio test. The analysis was performed using the tool Power and Sample Size (PSS) of the Statistical Analysis System software (SAS - version 9.4).

## Results

### Clinical characteristics and vitamin D levels in severely-symptomatic COVID-19 patients, mildly-symptomatic COVID-19 patients and non SARS-CoV-2 infected controls

The clinical characteristics of mildly-symptomatic COVID-19 patients, severely-symptomatic COVID-19 cases and non SARS-CoV-2 infected controls are reported in Table 1.

**Table 1:** Clinical characteristics of severely-symptomatic COVID-19 patients, mildly-symptomatic COVID-19 patients and non SARS-CoV-2 infected control subjects

	Whole cohort (***N*** = 361)	Non SARS-CoV-2 infected Control (***N*** = 206)	Mildly symptomatic COVID-19 (***n*** = 52)	Severely Symptomatic COVID-19 (***n*** = 103)	***P***-value
Age, years *median [IQR]*	66.00 [54.00–78.00]	67.00 [57.00–77.00]	57.50 [51.00–86.05]	66.70 [56.10–77.40]	0.649 ǂ
Male *N(%)*	243 (67%)	142 (69%)	31 (60%)	70 (68%)	0.435†
CV disease ^a^ *N(%)*	54 (15%)	31 (15%)	11 (21%)	12 (12%)	0.293†
At least 1 comorbidity ^b^ *N(%)*	181 (50%)	95 (46%)	23 (44%)	63 (61%)	0.03†
Obesity *N (%)*	67 (19%)	42 (20%)	5 (10%)	20 (19%)	0.196†
Diabetes *N (%)*	61 (17%)	31 (15%)	11 (21%)	19 (18%)	0.510†
Hypertension *N (%)*	140 (39%)	75 (36%)	13 (25%)	52 (51%)	0.005†
vitamin D deficiency (25OHD ≤20 ng/mL) *N(%)*	127 (35%)	57 (28%)	6 (12%)	64 (62%)	< 0.0001†
25OHD ng/mL *mean (SD)*	24.0 (10.6)	25.4 (9.4)	30.3 (8.4)	18.2 (11.4)	< 0.0001‡

*IQR* Interquartile range; *SD* Standard Deviation; *CV* Cardiovascular; ǂ = Kruskal-Wallis test; † = Chi square test; ‡ = ANOVA model;

^a^ cardiovascular disease: coronary heart disease, stroke, myocardial infarction; ^b^ at least 1 comorbidity among obesity, diabetes, arterial hypertension

**Fig. 2:**
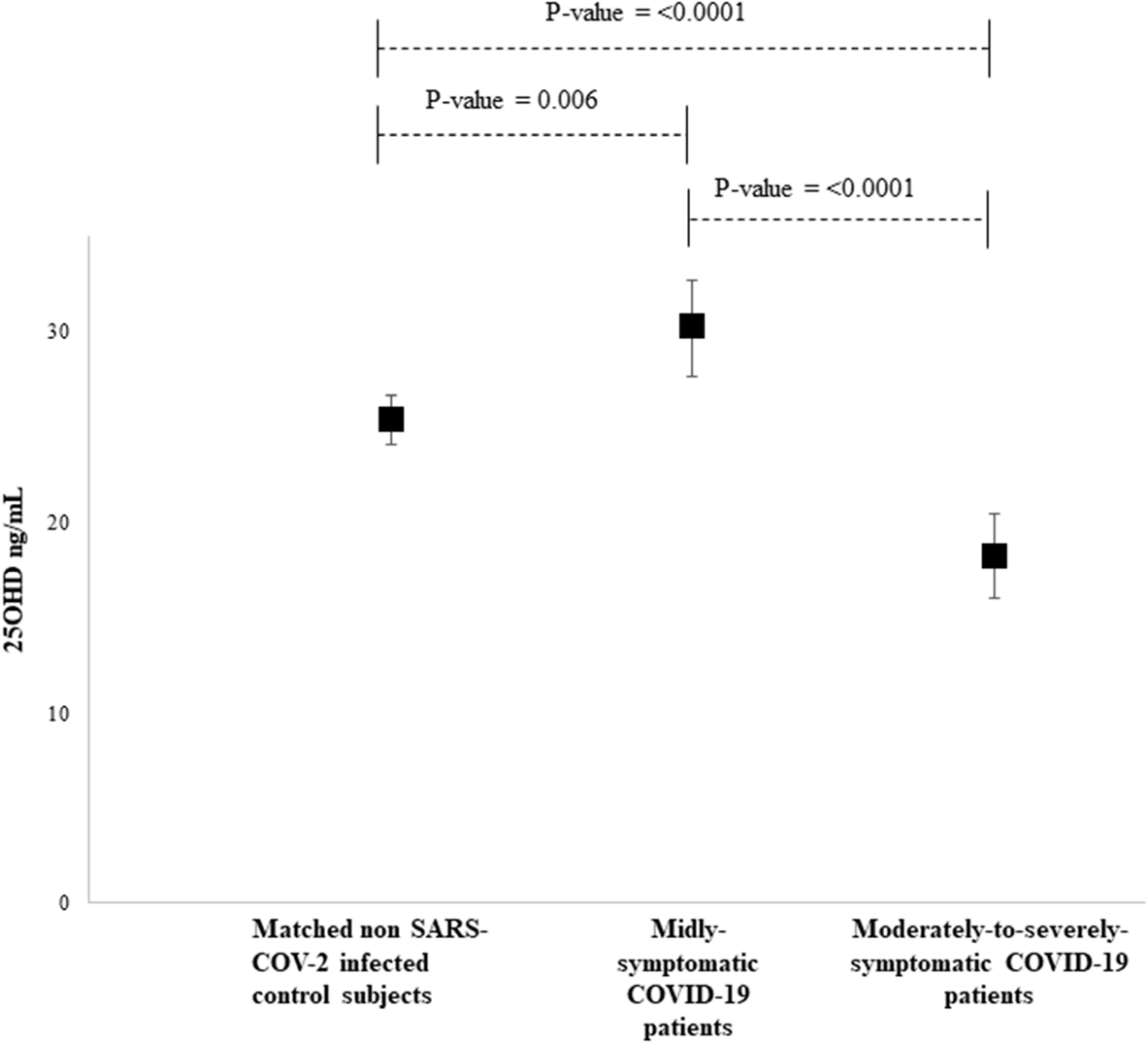
25-hydroxy-vitamin D levels in mildly-symptomatic COVID-19 patients, in severely-symptomatic COVID-19 patients and in matched non SARS-CoV-2 infected control subjects. Legend: Data are shown as mean 25-hydroxy-vitamin D values and their 95% confidence interval Tukey method is used to control the inflation of type I error due to head to head comparisonsP-value: SARS-CoV-2 infected control subjects vs mildly-symptomatic COVID-19 patients 0.006, SARS-CoV-2 infected control subjects vs severely-symptomatic COVID-19 patients < 0.0001, mildly-symptomatic COVID-19 patients vs severely-symptomatic COVID-19 patients < 0.0001

No difference was observed for age and gender and cardiovascular (CV) disease, while a higher prevalence of hypertension or at least one major comorbidity (among obesity, diabetes and hypertension) was observed in severely-symptomatic COVID-19 patients than in mildly-symptomatic COVID-19 cases and controls. Moreover, significant difference was observed for mean 25OHD levels among severely-symptomatic COVID-19 patients (18.2 ± 11.4 ng/mL), mildly-symptomatic COVID-19 patients (30.3 ± 8.4 ng/mL) and controls (25.4 ± 9.4 ng/mL). Head to head comparisons between mean levels of 25OHD showed significant lower values in severely-symptomatic COVID-19 patients respect to mildly-symptomatic COVID-19 patients and to non SARS-CoV-2 infected controls. Moreover, mildly-symptomatic COVID-19 cases showed significantly higher mean 25OHD values than non SARS-CoV-2 infected controls (Fig. 2). All comparisons are adjusted for age, gender, CV disease and each comorbidity.

Table 1 also shows a significant difference among the proportion of patients with vitamin D deficiency among the 3 groups (62% in severely-symptomatic COVID-19 patients, 12% in mildly-symptomatic COVID-19 patients and 28% in controls).

Concerning severely-symptomatic COVID-19 patients no statistically significant differences in 25OHD values were found between patients taking tocilizumab (12.5 ± 5.8 ng/mL), dexamethasone (18.8 ± 11.7 ng/mL) or chloroquine (16.3 ± 10.2 ng/mL), as compared to those not taking these drugs (19.5 ± 11.3 ng/mL, 18.7 ± 11.6 ng/mL, 18.8 ± 11.7 ng/mL, 20.3 ± 12.4 ng/mL, respectively, *p* > 0.1 for all comparisons). Similarly no statistically significant differences in IL-6 values were found between patients taking tocilizumab (34.5 ± 24.5 pg/mL), dexamethasone (38.0 ± 36.8 pg/mL) or chloroquine (39.8 ± 38.8 pg/mL), as compared to those not taking these drugs (42.0 ± 50.6 pg/mL, 40.2 ± 45.2 pg/mL, 40.2 ± 45.9 pg/mL, 39.6 ± 49.1 pg/mL, respectively, p > 0.1 for all comparisons).

### Vitamin D status and severe respiratory dysfunction requiring admission to ICU in severely-symptomatic COVID-19 patients

After hospital admission, 54 subjects (52.4%) were admitted to ICU, because of severe respiratory failure requiring continuous positive airway pressure and/or endo-tracheal intubation (Fig. 1). The presence of interstitial pneumonia was confirmed by computed tomography in all these cases. As shown in Table 2, these patients were more likely to be males, and affected with at least one major comorbidity. Concentrations of IL-6, lactate dehydrogenase (LDH) and neutrophils levels were higher, while 25OHD, lymphocytes and platelets levels were lower in patients subsequently admitted to ICU than in those without severe respiratory distress. Although severely-symptomatic COVID-19 patients admitted in ICU tended to have higher D-dimer levels than severely-symptomatic COVID-19 patients not admitted to ICU and were more frequently treated with tocilizumab (11.1% vs 4.1%, respectively *p* = 0.183), dexamethasone (27.8% vs 16.3%, respectively, *p* = 0.163) or chloroquine (57.4% vs 42.9%, *p* = 0.140), these differences were not statistically significant. In severely-symptomatic COVID-19 patients 25OHD levels were inversely associated with interleukin-6 (IL-6, ρ = − 0.282, *p*-value = 0.004) and directly with platelets levels (ρ = 0.178, p-value = 0.073).

**Table 2:** Clinical characteristics of severely-symptomatic COVID-19 patients admitted to hospital ward or to Intensive Care Unit

	Whole cohort (***N*** = 103)	Patients admitted to hospital ward (***n*** = 49)	Patients admitted to Intensive Care Unit (***n*** = 54)	***P***-value
Age, years *mean(SD)*	66.12 (14.14)	68.82 (14.93)	63.67 (13.00)	0.064 ‡
Males *N(%)*	70 (68%)	28 (57%)	42 (78%)	0.025†
CV diseases^a^ *N (%)*	12 (12%)	4 (8%)	8 (15%)	0.293†
At least 1 comorbidity^b^ *N(%)*	63 (61%)	25 (51%)	38 (70%)	0.044†
Obesity *N (%)*	20 (19%)	7 (14%)	13 (24%)	0.210†
Diabetes *N (%)*	19 (18%)	7 (14%)	12 (22%)	0.300†
Hypertension *N (%)*	52 (50%)	21 (43%)	31 (57%)	0.140†
25OHD *ng/mL mean(SD)*	18.2 (11.4)	22.4 (12.7)	14.4 (8.6)	0.0003‡
vitamin D deficiency (25OHD ≤20 ng/mL) *N(%)*	64 (62%)	22 (45%)	42 (78%)	0.0006†
Creatinine μmol/L *median [IQR]*	0.86 [0.72–1.04]	0.93 [0.81–1.04]	0.80 [0.66–0.95]	0.008ǂ
IL-6 pg/mL *median [IQR]*	25.00 [13.00–51.00]	16.00 [8.00–32.00]	42.95 [19.00–56.00]	0.0002ǂ
D-Dimer ng/mL *median [IQR]*	2482 [1697–3267]	2118 [1125–3110]	2786 [1585–3986]	0.403
C-reactive Protein, nmol/L *median [IQR]*	2.16 [0.78–8.21]	2.67 [0.88–5.82]	1.81 [0.72–9.83]	0.872ǂ
Fibrinogen, g/L *mean(SD)*	482.3 (167.4)	503.6 (144.4)	463.0 (181.5)	0.222‡
Lactate dehydrogenase, mU/mL *median [IQR]*	260.00 [230.00–312.00]	253.00 [222.00–279.00]	284.50 [238.00–364.00]	0.009ǂ
Leukocytes count, ×10^9^/L *median [IQR]*	7.90 [5.50–10.10]	7.00 [5.20–8.30]	8.50 [6.10–10.80]	0.052ǂ
Neutrophils count, × 10^9^/L *median [IQR]*	5.60 [3.30–7.40]	3.90 [3.10–5.70]	6.70 [3.80–8.00]	0.002ǂ
Lymphocytes count, ×10^9^/L *median [IQR]*	1.50 [1.00–2.00]	1.50 [1.30–2.20]	1.30 [0.90–1.90]	0.039ǂ
Platelets count, ×10^9^/L *median [IQR]*	283.00 [214.00–369.00]	330.00 [276.00–399.00]	228.00 [186.00–345.00]	0.0005ǂ

*P*-value are referred to the comparison between patients admitted to Intensive Care Unit and those admitted to hospital ward

^a^ cardiovascular disease: coronary heart disease, stroke, myocardial infarction; ^b^ at least 1 comorbidity among obesity, diabetes, arterial hypertension. *SD* Standard Deviation, *IQR* interquartile range, ‡ = T-test, ǂ = Wilcoxon test, † = Chi-square test

Patients in intensive care unit: patients with respiratory distress requiring continuous positive airway pressure and/or endo-tracheal intubation. *25OHD* 25hydroxyvitamin D; *IL-6* interleukin-6

Lactate dehydrogenase normal values: 8–300 mU/mL; Fibrinogen normal values: 2.0–4.0 mg/dL; Leukocyte normal values: 4.5–11.0 × 109/L; Neutrophil normal values: 1.5–8.0 × 109/L; Absolute Lymphocyte Count normal values: 0.9–2.9 × 109/L; Platelet normal values: 150–400 × 109/L

The prevalence rates of vitamin D deficiency were significantly higher in patients requiring ICU admission than in those who did not (Table 2). ICU admission was inversely associated with 25OHD levels controlling for age, gender, creatinine, IL-6, LDH, neutrophil cell, lymphocytes and platelets levels and the presence of at least one major comorbidity (Table 3). A 1 ng/mL increase in 25OHD levels was associated with a reduction of 1% (95%CI 0 to 2% p –value = 0.011) of ICU admission risk.

**Table 3:** Factors independently associated with admission to intensive care Unit in severely-symptomatic COVID-19 patients

	RR (95% CI)	***P***-value
Age	0.993 (0.980 to 1.007)	0.325
Gender		
Female	Ref	
Male	1.222 (0.793 to 1.882)	0.363
At least 1 comorbidity ^a^		
No	Ref.	
Yes	1.556 (1.079 to 2.244)	0.018
Creatinine	0.503 (0.268 to 0.944)	0.032
IL-6	1.000 (0.997 to 1.004)	0.907
Lactate dehydrogenase	1.000 (1.000 to 1.001)	0.459
Neutrophil cells count	1.076 (1.018 to 1.136)	0.009
Lymphocytes count	0.860 (0.651 to 1.137)	0.289
Platelets count	0.998 (0.996 to 1.000)	0.089
25OHD	0.989 (0.981 to 0.997)	0.011

Patients in Intensive Care Unit: patients with respiratory distress requiring continuous positive airway pressure and/or endo-tracheal intubation. *25OHD* 25hydroxyvitamin D; *IL-6* interleukin-6;

^a^ at least 1 comorbidity among obesity, diabetes, arterial hypertension

### Vitamin D status and COVID-19 mortality in severely-symptomatic COVID-19 patients

After a mean of 19.1 ± 8.7 days of hospital stay (range 3–36 days) 19 patients (18.4%) died, all of them due to ARDS (Fig. 1). These subjects were older and showed significantly lower 25OHD levels, lymphocytes and platelets levels and higher IL-6, C-reacting protein (CRP), LDH and neutrophil levels than COVID-19 patients who survived (Table 4). The prevalence of diabetes was also significantly higher among deceased patients, while there were no differences for the presence of other comorbidities. Although deceased, severely-symptomatic COVID-19 patients tended to have higher D-dimer levels than severely-symptomatic COVID-19 patients who survived, this difference was not statistically significant. Likewise, the use of tocilizumab, dexamethasone and chloroquine was not different between patients who deceased and those who survived.

**Table 4:** Clinical characteristics of severely-symptomatic COVID-19 patients who survived and of COVID-19 patients who died in hospital

	Whole cohort (N = 103)	Surviving patients (***n*** = 84)	Non-surviving patients (***n*** = 19)	***P***-value
Age, years *mean(SD)*	66.12 (14.14)	64.72 (14.72)	72.32 (8.95)	0.006‡
Males *N(%)*	70 (68%)	57 (68%)	13 (68%)	0.962†
CV diseases^a^ *N(%)*	12 (12%)	11 (13%)	1 (5%)	0.458¥
At least 1 comorbidity^b^ *N(%)*	63 (61%)	48 (57%)	15 (79%)	0.078†
Obesity *N (%)*	20 (19%)	18 (21%)	2 (11%)	0.353¥
Diabetes *N (%)*	19 (18%)	10 (12%)	9 (47%)	0.001¥
Hypertension *N (%)*	52 (50%)	40 (48%)	12 (63%)	0.221†
25OHD *ng/mL mean(SD)*	18.2 (11.5)	19.3 (12.0)	13.2 (6.4)	0.0003‡
vitamin D deficiency (25OHD ≤20 ng/mL) *N(%)*	64 (62%)	51 (61%)	13 (68%)	0.532†
Creatinine μmol/L *median [IQR]*	0.86 [0.72–1.04]	0.87 [0.74–1.03]	0.75 [0.62–1.25]	0.284ǂ
IL-6 pg/mL *median [IQR]*	25.00 [13.00–51.00]	21.00 [10.45–45.90]	45.00 [28.00–99.00]	0.002ǂ
D-dimer ng/mL *median [IQR]*	2482 [1697–3267]	2134 [1303–2965]	3947 [1782–6223]	0.08 ǂ
C-reactive Protein, nmol/L *median [IQR]*	2.16 [0.78–8.21]	1.81 [0.69–5.55]	11.30 [4.78–21.71]	< 0.0001ǂ
Fibrinogen, g/L *mean(SD)*	482.3 (167.4)	475.5 (151.6)	512.6 (227.4)	0.505‡
Lactate dehydrogenase, mU/mL *median [IQR]*	260.00 [230.00–312.00]	252.00 [215.00–294.00]	330.00 [285.00–643.00]	< 0.0001ǂ
Leukocytes count, ×109/L *median [IQR]*	7.90 [5.50–10.10]	7.80 [5.35–9.80]	8.60 [6.40–12.30]	0.136ǂ
Neutrophils count, × 109/L *median [IQR]*	5.60 [3.30–7.40]	4.50 [3.20–6.80]	7.20 [5.60–9.90]	0.002ǂ
Lymphocytes count, ×109/L *median [IQR]*	1.50 [1.00–2.00]	1.50 [1.15–2.10]	1.10 [0.80–1.70]	0.029ǂ
Platelets count, ×109/L *median [IQR]*	299.00 [228.00–378.50]	299.00 [228.00–378.50]	213.00 [139.00–308.00]	0.0015ǂ

*P*-value are referred to the comparison between non-surviving patients and surviving patients

^a^cardiovascular disease: coronary heart disease, stroke, myocardial infarction; ^b^ at least 1 comorbidity: among obesity, diabetes, arterial hypertension. *SD* Standard Deviation, *IQR* interquartile range, ‡ = T-test, ǂ = Wilcoxon test, ¥ = Fisher test; † = Chi-square test

Lactate dehydrogenase normal values: 8–300 mU/mL; Fibrinogen normal values: 2.0–4.0 mg/dL; Leukocyte normal values: 4.5–11.0 × 10^9^/L; Neutrophil normal values: 1.5–8.0 × 10^9^/L; Absolute Lymphocyte Count normal values: 0.9–2.9 × 10^9^/L; Platelet normal values: 150–400 × 10^9^/L

Mortality was inversely associated with 25OHD levels controlling for age, gender, diabetes, IL-6, CRP, LDH, neutrophil cell, lymphocytes, and platelets levels (Table 5). A 1 ng/mL increase in 25OHD levels was associated with a reduction of 4% (95%CI 1 to 6% p –value = 0.002) of death risk. All severely-symptomatic COVID-19 patients who survived were discharged after a mean ± SD hospital stay of 16.3 ± 8.0 and 21.0 ± 9.1 days, respectively, for those admitted to a ward and those in need of ICU.

**Table 5:** Factors independently associated with in-hospital mortality in severely-symptomatic COVID-19 patients

	RR (95% CI)	***P***-value
Age	1.070 (1.029 to 1.114)	0.0008
Gender		
Female	Ref	0.369
Male	1.672 (0.545 to 5.126)	
Diabetes		
No	Ref.	< 0.0001
Yes	3.446 (1.900 to 6.248)	
IL-6	1.009 (1.002 to 1.017)	0.019
C-reactive Protein	1.035 (1.001 to 1.069)	0.042
Lactate dehydrogenase	1.004 (1.001 to 1.006)	0.006
Neutrophil cells count	1.064 (0.936 to 1.209)	0.344
Lymphocytes count	0.437 (0.204 to 0.938)	0.034
Platelets count	1.002 (0.997 to 1.006)	0.487
25OHD	0.961 (0.937 to 0.985)	0.002

**Fig. 3:**
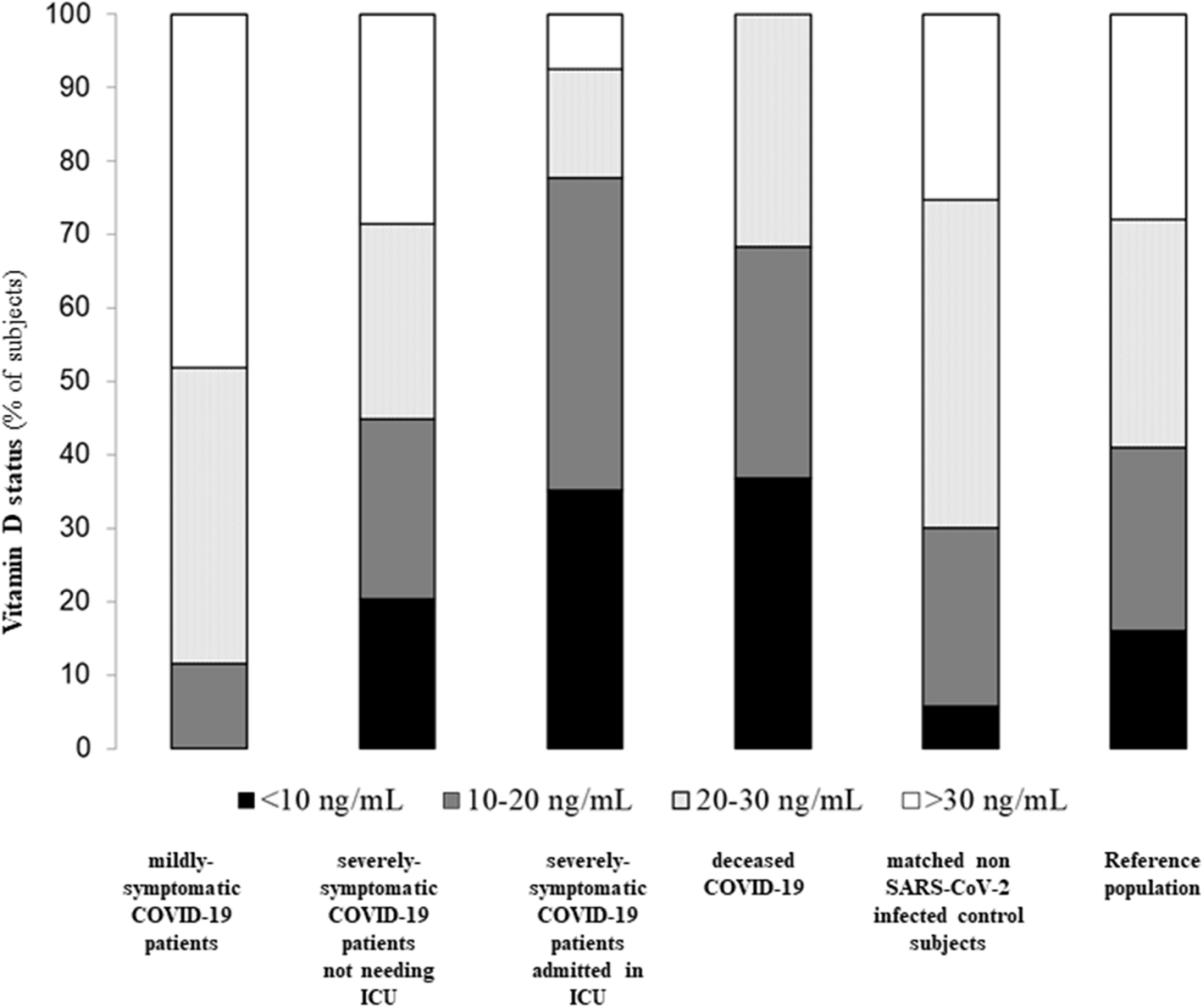
Prevalence rates of vitamin D insufficiency or deficiency and of severe hypovitaminosis D in mildly-symptomatic SARS-CoV-2 infected subjects (mildly-symptomatic COVID-19), in COVID-19 patients admitted to hospital ward (COVID-19 non-ICU), in COVID-9 patients admitted to intensive care unit (COVID-19 ICU), in COVID-19 patients who died COVID-19 deceased) and in control subjects (matched controls). Legend: Data are shown as prevalence rates of vitamin D insufficiency (25OHD < 30 ng/mL), deficiency (25OHD < 20 ng/mL), or severe hypovitaminosis D (25OHD < 10 ng/mL) (B). ICU: intensive care Unit. Reference population: 3174 consecutive subjects, who underwent 25OHD measurement in Siena (Italy) between January 01 and March 31, 2020 in the frame of a routine health check with no epidemiological and clinical evidence of SARS-CoV-2 infection

*25OHD* 25hydroxyvitamin D; *IL-6* interleukin-6;

The proportions of patients grouped by different thresholds of vitamin D levels (< 10 ng/mL; 10–20 ng/mL, 20–30 mg/mL and > 30 ng/mL) for the mildly-symptomatic COVID-19 patients, severely-symptomatic COVID-19 patients not needing ICU, severely-symptomatic COVID-19 patients admitted in ICU, deceased severely-symptomatic COVID-19 patients, non SARS-CoV-2 infected controls and the reference population (Siena’s cohort as the source of our control group) are depicted in Fig. 3. As is evident, the prevalence of both vitamin D 25OHD < 10 ng/mL and 25OHD between 10 and 20 ng/mL was higher in severely COVID-19 cases (considered as overall group as well as in ICU-admitted and deceased cases) than in mildly-symptomatic COVID-19 patients or controls, respectively.

## Discussion

The dramatic evolution of SARS-CoV-2 pandemic, along with its higher morbidity and mortality rates, urged us to investigate the possible mechanisms leading to severe clinical outcomes in the attempt to identify effective treatments [1]. Within this context, we measured 25OHD levels in a series of SARS-CoV-2 infected patients from the first pandemic flow, including either mildly-symptomatic subjects or severely symptomatic COVID-19 cases, the latter admitted to hospital because of different degrees of respiratory insufficiency. An age and sex matched group of uninfected subjects was also recruited as reference.

In the overall sample, circulating 25OHD levels were lower in hospitalized, severely-symptomatic COVID-19 patients than in midly-symptomatic-COVID-19 subjects and in non SARS-CoV-2 infected controls. Moreover, in severely-symptomatic COVID-19 patients 25OHD levels at hospital admission predicted both the severity of the respiratory distress (as mirrored by the needing of ICU admission) and the mortality. Overall, these data confirm previous similar studies [16–22] and demonstrate on a prospective setting that vitamin D deficiency is associated with the clinical outcomes of COVID-19, independently of inflammatory markers (e.g. IL-6 and CRP), age, or the presence of major comorbidities such as obesity, diabetes and hypertension, that are commonly observed among hospitalized COVID-19 patients.

Likewise, different studies suggested a possible association between 25OHD levels and COVID-19 on the basis of indirect evidences only, such as the relationship between the risk of SARS-CoV-2 infection and latitude [14] or the occurrence of an inverse correlation between mean vitamin D levels and the number of COVID-19 cases in European countries [15]. Interestingly, other larger observations, including cohort studies, reported the presence of significantly lower vitamin D levels in SARS-CoV-2 infected subjects compared with SARS-CoV-2 negative patients, thus suggesting a relationship between insufficient vitamin D levels and SARS-Cov-2 positivity [25, 26].

Our data, therefore, are in line with most of the above observations, potentially suggesting that reduced vitamin D levels may favor SARS-Cov-2 infection and may have a role in the risk of severe respiratory dysfunction and mortality during the course of hospitalization in patients affected by COVID-19. The observed inverse association between circulating 25OHD and IL-6, a cytokine involved in inflammatory response and autoimmune diseases, contributes to characterize, at least in part, the possible role exerted by vitamin D in this disease. Indeed, IL-6 is crucial in regulating the inflammatory response, and increased IL-6 levels in COVID-19 patients have been repeatedly related to the severity and prognosis of this disease [4, 5]. Consistent with this hypothesis, tocilizumab, a recombinant humanized antihuman IL-6 receptor monoclonal antibody, has been widely used with the aim to improve the clinical outcome and possibly reduce mortality in severe and critical COVID-19 patients [27–29]. Interestingly, we previously demonstrated that a low vitamin D status is associated with an enhanced acute inflammatory reaction in patients undergoing bisphosphonate treatment, that becomes particularly relevant when 25OHD levels are below 20 ng/mL [30]. Vitamin D supplementation in those patients was able to reduce IL-6 levels and to decrease the prevalence of such inflammatory response. Importantly, while tocilizumab non-selectively blocks both anti-inflammatory and pro-inflammatory actions of IL-6, vitamin D does not target IL-6 receptors and thus directly reduces IL-6 production from immune cells avoiding potential deleterious effects related to the complete suppression of the anti-inflammatory actions of IL-6 [31].

In addition, previous experimental studies demonstrated that vitamin D is locally activated in lung tissues and may exert a preventive effect on experimental interstitial pneumonia [32]. This might occur through different mechanisms that not only include the suppression of the pro-inflammatory cytokine production, but also the modulation of neutrophil activity (thereby reducing their excessive activation and recruitment into inflamed lung that is in part responsible of alveolar damage) and the killing of enveloped viruses through induction of cathelicidin and defensins [7, 15].

Moreover, vitamin D protected the pulmonary vascular barrier from acute inflammatory injury in mice by locally targeting the renin-angiotensin system [33], whose dysregulation has been implicated in favoring the SARS-CoV-2 entry into alveolar cells with massive cytokine activation and development of ARDS [2, 34]. Additional positive effects of vitamin D at the lungs level also include the stimulation of surfactant synthesis by alveolar type-II cells, thus improving alveolar surface tension and exerting a protective role against inflammation and oxidative stress [35]. These data suggest that an adequate vitamin D status might have potentially favorable effects on the clinical outcome of COVID-19, by improving the pulmonary vascular barrier and limiting the cytokine storm.

The hypothesis of a role for vitamin D deficiency in favoring the COVID-19 related respiratory dysfunction is further supported by epidemiologic data showing that the mortality rate of COVID-19 infection between the onset of the first pandemic flow and the summer time was higher (> 10%) in southern European countries [13–15], such as Italy and Spain, in which vitamin D deficiency is known to be highly prevalent in the winter season, likely due to the lack of fortification and supplementation policies [36]. Indeed, the SARS-CoV-2 pandemic, between January and May 2020, has shown a larger diffusion in the Northern hemisphere along the 30–50°N latitude zone, while COVID-19 related mortality, particularly concerning white Caucasian subjects, importantly decreased (around 2–6%) in most countries below the 37°N parallel [37], where the sunlight exposure allows to produce adequate vitamin D levels even in fall and winter seasons [6]. Likewise, death rates have been lower (well below 5%) in other Countries of Caucasian ancestry from the Southern hemisphere such as Australia and New Zealand, in concomitance with the local summer season [38], while have remarkably decreased in our country and other European countries after the wintertime. The more recent pandemic flows in the Northern hemisphere mostly recapitulated the above observations, showing a progressive increase in mortality rates from October–November 2020 onwards. In keeping with these findings, in US COVID-19 mortality rates have been remarkably higher (up to 4–5 times) among black American patients, that generally have lower 25OHD concentrations than the other ethnic groups [39].

In our study, 25OHD levels were higher in mildly-symptomatic COVID-19 cases than in non SARS-CoV-2 controls. Although, a selection bias cannot be totally excluded, this finding could be explained by the large number of subjects taking cholecalciferol supplements (i.e. about 79%) among mildly-symptomatic COVID-19 cases, a percentage much higher than that reported in the healthy Italian population (40.5 and 12% among adult women and men, respectively) [40]. Moreover, such an observation is also consistent with the study results showing an inverse relationship between COVID-19 severity and 25OHD status.

Importantly, since we measured 25OHD levels at the time SARS-Cov-2 infection we cannot completely rule out the possibility that our findings could be due to reverse causality, i.e. that the acute illness could have led to a reduction in total 25OHD levels via a vitamin D binding protein suppression or other mechanisms. Indeed, whether or not free 25OHD levels are a better predictor than total 25OHD levels for health outcomes is still controversial [41]. Likewise, as 25OHD levels were measured when patients were admitted to the hospital, the finding of lower 25OHD in COVID-19 hospitalized patients may be expected, in view of the fact that vitamin D is considered an acute phase reactant and might decrease in case of severe acute infection [42, 43]. In this respect, however, most of the studies that assessed the relationship between 25OHD levels measured before SARS-Cov-2 infection, evidenced a similar association between vitamin D deficiency and the severity of COVID-19 disease [26, 44–46]. These include a very recent update from the large UK Biobank cohort (*n* = 353,299 participants with 1082 SARS-Cov-2 affected cases) showing a significantly positive association between vitamin D insufficiency (as assessed in the baseline visit performed between 2006 and 2010) and COVID-19 hospitalization or severity [46], thus disconfirming previous information derived from the same cohort with a much lower number of SARS-Cov-2 affected cases [47–49]. In that case reverse-causality is excluded.

Additional limitations of our study include the relatively small sample of COVID-19 cases and the choice of a group of mildly-symptomatic patients, who were nursing home residents or staff, and, therefore, demographically distinct from the patients. The inclusion of such a group, however, gave us the possibility to have a further reference group. Indeed, the finding that the mildly-symptomatic patients and control subjects had higher and more frequently normal 25OHD levels as compared with COVID-19 patients showing respiratory insufficiency (Fig. 2), may strengthen the hypothesis of a causative role of vitamin D in modulating the progression from mildly-symptomatic COVID-19 towards a clinically overt COVID-19 disease with variable severity. Moreover, the choice of including data from a non SARS-CoV-2 infected control group, derived from the large and unbiased reference sample of subjects from the same population undergoing 25OHD measurement during the same period of time (i.e. January 01–March 31, 2020), allowed us to obtain a representative picture about vitamin status in our Country during the SARS-CoV-2 outbreak. Since the latter group included either hospitalized patients or community dwelling individuals a selective pressure is unlikely, thus confirming that vitamin D deficiency is common among the general population in our Country in the winter season. Although these control individuals did not show epidemiological and clinical evidence of SARS-CoV-2 infection at the time of 25OHD measurement, however, we have no information regarding whether or not these individuals got infected thereafter. Finally, the lack of measurement of vitamin D levels with the gold standard method (i.e. Liquid Chromatography Mass Spectrometry) has to be considered a further study limit, even though the method we used is still considered reliable for the routine clinical purposes.

## Conclusions

Notwithstanding all the above limitations, in our sample of hospitalized COVID-19 patients with respiratory failure we detected a remarkably high prevalence of vitamin D deficiency. Importantly, low 25OHD levels at hospital admission were associated with increased IL-6 levels and predicted both the severity of respiratory distress and mortality during the course of hospitalization, independently of other comorbidities. Therefore, these data deserve interest since, even without considering the possible causal link between vitamin D deficiency and the clinical course of COVID-19, the finding of low 25OHD levels in patients with severe COVID-19 could be at least considered as a useful prognostic marker (with a greater predictive capacity than IL-6 or CRP). In addition, our observations, together with the low cost and the lack of safety concerns related to vitamin D supplementation [50], further support the need of adequately powered and well designed intervention trials aimed at exploring whether vitamin D replacement therapy might prevent the risk of respiratory failure in patients with SARS-CoV-2 infection [51], as suggested by some, but not all, preliminary observations [52–56].

## Data Availability

The datasets used and/or analysed during the current study are available from the corresponding author on reasonable request.

## References

[1] Cook DJ, Marshall JC, Fowler RA (2020). Critical illness in patients with COVID-19: mounting an effective clinical and research response. JAMA.

[2] Sarzi-Puttini P, Giorgi V, Sirotti S, Marotto D, Ardizzone S, Rizzardini G (2020). COVID-19, cytokines and immunosuppression: what can we learn from severe acute respiratory syndrome?. Clin Exp Rheumatol.

[3] Mehta P, McAuley DF, Brown M, Sanchez E, Tattersall RS, Manson JJ, HLH Across Speciality collaboration, UK, HLH across specialty collaboration, UK (2020). COVID-19: consider cytokine storm syndromes and immunosuppression. Lancet.

[4] McGonagle D, Sharif K, O'Regan A, Bridgewood C (2020). The Role of Cytokines including Interleukin-6 in COVID-19 induced Pneumonia and Macrophage Activation Syndrome-Like Disease. Autoimmun Rev.

[5] Cummings MJ, Baldwin MR, Abrams D, Jacobson SD, Meyer BJ, Balough EM (2020). Epidemiology, clinical course, and outcomes of critically ill adults with COVID-19 in new York City: a prospective cohort study. Lancet.

[6] Bouillon R, Marcocci C, Carmeliet G, White JH, Dawson-Hughes B, Lips P (2019). Skeletal and Extraskeletal actions of vitamin D: current evidence and outstanding questions. Endocr Rev.

[7] Quesada-Gomez JM, Entrenas-Castillo M, Bouillon R (2020). Vitamin D receptor stimulation to reduce acute respiratory distress syndrome (ARDS) in patients with coronavirus SARS-CoV-2 infections: revised Ms SBMB 2020_166. J Steroid Biochem Mol Biol.

[8] Martineau AR, Jolliffe DA, Hooper RL, Greenberg L, Aloia JF, Bergman P (2017). Vitamin D supplementation to prevent acute respiratory tract infections: systematic review and meta-analysis of individual participant data. Br Med J.

[9] Jolliffe DA, Camargo CA, Sluyter JD, Aglipay M, Aloia JF, Ganmaa D (2021). Vitamin D supplementation to prevent acute respiratory infections: a systematic review and meta-analysis of aggregate data from randomised controlled trials. Lancet Diabetes Endocrinol.

[10] Havers F, Smeaton L, Gupte N, Detrick B, Bollinger RC, Hakim J, Kumarasamy N, Andrade A, Christian P, Lama JR, Campbell TB, Gupta A, ACTG PEARLS, NWCS 319 Study Teams (2014). 25-Hydroxyvitamin D insufficiency and deficiency is associated with HIV disease progression and virological failure post-antiretroviral therapy initiation in diverse multinational settings. J Infect Dis.

[11] The Scientific Advisory Committee on Nutrition (SACN) rapid review on vitamin D and acute respiratory tract infections (ARTI). December 2020. https://www.gov.uk/government/publications/sacn-rapid-review-vitamin-d-and-acute-respiratory-tract-infections Last .

[12] Mitchell F (2020). Vitamin-D and COVID-19: do deficient risk a poorer outcome?. Lancet Diabetes Endocrinol.

[13] Grant WB, Lahore H, McDonnell SL, Baggerly CA, French CB, Aliano JL (2020). Evidence that vitamin D supplementation could reduce risk of influenza and COVID-19 infections and deaths. Nutrients.

[14] Rhodes JM, Subramanian S, Laird E, Kenny RA (2020). Editorial: low population mortality from COVID-19 in countries south of latitude 35 degrees north supports vitamin D as a factor determining severity. Aliment Pharmacol Ther.

[15] Ilie PC, Stefanescu S, Smith L (2020). The role of vitamin D in the prevention of coronavirus disease 2019 infection and mortality. Aging Clin Exp Res.

[16] Panagiotou G, Tee SA, Ihsan Y, Athar W, Marchitelli G, Kelly D, Boot CS, Stock N, Macfarlane J, Martineau AR, Burns G, Quinton R (2020). Low serum 25-hydroxyvitamin D (25[OH]D) levels in patients hospitalised with COVID-19 are associated with greater disease severity. Clin Endocrinol.

[17] Hars M, Mendes A, Serratrice C, Herrmann FR, Gold G, Graf C (2020). Sex-specific association between vitamin D deficiency and COVID-19 mortality in older patients. Osteoporos Int.

[18] Radujkovic A, Hippchen T, Tiwari-Heckler S, Dreher S, Boxberger M, Merle U (2020). Vitamin D deficiency and outcome of COVID-19 patients. Nutrients..

[19] De Smet D, De Smet K, Herroelen P, Gryspeerdt S, Martens GA (2021). Serum 25(OH)D level on hospital admission associated with COVID-19 stage and mortality. Am J Clin Pathol.

[20] Hernández JL, Nan D, Fernandez-Ayala M, García-Unzueta M, Hernández-Hernández MA, López-Hoyos M, Muñoz-Cacho P, Olmos JM, Gutiérrez-Cuadra M, Ruiz-Cubillán JJ, Crespo J, Martínez-Taboada VM (2021). Vitamin D status in hospitalized patients with SARS-CoV-2 infection. J Clin Endocrinol Metab.

[21] Merzon E, Tworowski D, Gorohovski A, Vinker S, Golan Cohen A, Green I, Frenkel-Morgenstern M (2020). Low plasma 25(OH) vitamin D level is associated with increased risk of COVID-19 infection: an Israeli population-based study. FEBS J.

[22] Maghbooli Z, Sahraian MA, Ebrahimi M, Pazoki M, Kafan S, Tabriz HM, Hadadi A, Montazeri M, Nasiri M, Shirvani A, Holick MF (2020). Vitamin D sufficiency, a serum 25-hydroxyvitamin D at least 30 ng/mL reduced risk for adverse clinical outcomes in patients with COVID-19 infection. PLoS One.

[23] Duca A, Piva S, Focà E, Latronico N, Rizzi M (2020). Calculated Decisions: Brescia-COVID Respiratory Severity Scale (BCRSS)/Algorithm. Emerg Med Pract.

[24] Zou G (2004). A modified Poisson regression approach to prospective studies with binary data. Am J Epidemiol.

[25] Kaufman HW, Niles JK, Kroll MH, Bi C, Holick MF (2020). SARS-CoV-2 positivity rates associated with circulating 25-hydroxyvitamin D levels. PLoS One.

[26] Meltzer DO, Best TJ, Zhang H, Vokes T, Arora V, Solway J (2020). Association of Vitamin D Status and Other Clinical Characteristics with COVID-19 test results. JAMA Netw Open.

[27] Xu X, Han M, Li T, Wang D, Fu B, Zhou Y (2020). Effective treatment of severe COVID-19 patients with tocilizumab. Proc Natl Acad Sci U S A.

[28] Capra R, De Rossi N, Mattioli F, Romanelli G, Scarpazza C, Sormani MP (2020). Impact of low dose tocilizumab on mortality rate in patients with COVID-19 related pneumonia. Eur J Intern Med.

[29] Ghosn L, Chaimani A, Evrenoglou T, Davidson M, Graña C, Schmucker C (2021). Interleukin-6 blocking agents for treating COVID-19: a living systematic review. Cochrane Database Syst Rev.

[30] Merlotti D, Rendina D, Muscariello R, Picchioni T, Alessandri M, De Filippo G (2020). Preventive role of vitamin D supplementation for acute phase reaction after bisphosphonate infusion in Paget's disease. J Clin Endocrinol Metab.

[31] Silberstein M (2021). COVID-19 and IL-6: why vitamin D (probably) helps but tocilizumab might not. Eur J Pharmacol.

[32] Tsujino I, Ushikoshi-Nakayama R, Yamazakj T, Matsumoto N, Saito I (2019). Pulmonary activation of vitamin D3 and preventive effect against interstitial pneumonia. J Clin Biochem Nutr.

[33] Kong J, Zhu X, Shi Y, Liu T, Chen Y, Bhan I, Zhao Q, Thadhani R, Li YC (2013). VDR attenuates acute lung injury by blocking Ang-2-Tie-2 pathway and renin-angiotensin system. Mol Endocrinol.

[34] Hoffmann M, Kleine-Weber H, Schroeder S, Krüger N, Herrler T, Erichsen S, Schiergens TS, Herrler G, Wu NH, Nitsche A, Müller MA, Drosten C, Pöhlmann S (2020). SARS-CoV-2 cell entry depends on ACE2 and TMPRSS2 and is blocked by a clinically proven protease inhibitor. Cell.

[35] Phokela SS, Peleg S, Moya FR, Alcorn JL (2005). Regulation of human pulmonary surfactant protein gene expression by 1alpha,25- dihydroxyvitamin D3. Am J Physiol Lung Cell Mol Physiol.

[36] Lips P, Cashman KD, Lamberg-Allardt C, Bischoff-Ferrari HA, Obermayer-Pietsch B, Bianchi ML (2019). Current vitamin D status in European and Middle East countries and strategies to prevent vitamin D deficiency: a position statement of the European calcified tissue society. Eur J Endocrinol.

[37] COVID-19 Dashboard by the Center for Systems Science and Engineering (CSSE) at Johns Hopkins University. Last ; https://coronavirus.jhu.edu/map.html; 10.1016/S1473-3099(22)00434-0 PMC9432867 36057267

[38] Coronavirus (COVID-19) health alert. Australian Government, Department of Health. https://www.health.gov.au/resources/collections/coronavirus-covid-19-at-a-glance-infographic-collection#collection-description Last ;

[39] The Color of Coronavirus: COVID-19 Deaths by Race and Ethnicity in the U.S. https://www.apmresearchlab.org/covid/deaths-by-race#black Last .

[40] https://www.aifa.gov.it/-/vitamina-d-consumi-e-spesa-ridotti-dall-introduzione-della-nota-96.

[41] Bouillon R, Schuit F, Antonio L, Rastinejad F (2020). Vitamin D binding protein: a historic overview. Front Endocrinol (Lausanne).

[42] Reid D, Knox S, Talwar D, O'Reilly DJ, Blackwell S, Kinsella J (2010). Acute changes in the systemic inflammatory response is associated with transient decreases in circulating 25-hydroxyvitamin D concentrations following elective knee arthoplasty. Ann Clin Biochem.

[43] Louw JA, Werbeck A, Louw ME, Kotze TJ, Cooper R, Labadarios D (1992). Blood vitamin concentrations during the acute-phase response. Crit Care Med.

[44] Charoenngam N, Shirvani A, Reddy N, Vodopivec DM, Apovian CM, Holick MF (2021). Association of Vitamin D Status with Hospital Morbidity and Mortality in adult hospitalized patients with COVID-19. Endocr Pract.

[45] Demir M, Demir F, Aygun H (2021). Vitamin D deficiency is associated with COVID-19 positivity and severity of the disease. J Med Virol.

[46] Li S, Cao Z, Yang H, Zhang Y, Xu F, Wang Y (2021). Metabolic healthy obesity, vitamin D status, and risk of COVID-19. Aging Dis.

[47] Hastie CE, Mackay DF, Ho F, Celis-Morales CA, Katikireddi SV, Niedzwiedz CL, Jani BD, Welsh P, Mair FS, Gray SR, O’Donnell CA, Gill JMR, Sattar N, Pell JP (2020). Vitamin D concentrations and COVID-19 infection in UK biobank. Diabetes Metab Syndr.

[48] Raisi-Estabragh Z, McCracken C, Bethell MS, Cooper J, Cooper C, Caulfield MJ (2020). Greater risk of severe COVID-19 in Black, Asian and Minority Ethnic populations is not explained by cardiometabolic, socioeconomic or behavioural factors, or by 25(OH)-vitamin D status: study of 1326 cases from the UK Biobank. J Public Heal. (Oxf).

[49] Hastie CE, Pell JP, Sattar N (2020). Vitamin D and COVID-19 infection and mortality in UK biobank. Eur J Nutr.

[50] Bouillon R (2020). Safety of High-Dose Vitamin D Supplementation. J Clin Endocrinol Metab.

[51] Camargo CA, Martineau AR (2020). Vitamin D to prevent COVID-19: recommendations for the design of clinical trials. FEBS J.

[52] Entrenas Castillo M, Entrenas Costa LM, Vaquero Barrios JM, Alcalá Díaz JF, López Miranda J, Bouillon R, Quesada Gomez JM (2020). Effect of calcifediol treatment and best available therapy versus best available therapy on intensive care unit admission and mortality among patients hospitalized for COVID-19: a pilot randomized clinical study. J Steroid Biochem Mol Biol.

[53] Cangiano B, Fatti LM, Danesi L, Gazzano G, Croci M, Vitale G (2020). Mortality in an Italian nursing home during COVID-19 pandemic: correlation with gender, age, ADL, vitamin D supplementation, and limitations of the diagnostic tests. Aging (Albany NY).

[54] Annweiler G, Corvaisier M, Gautier J, Dubée V, Legrand E, Sacco G, Annweiler C (2020). Vitamin D supplementation associated to better survival in hospitalized frail elderly COVID-19 patients: the GERIA-COVID quasi-experimental study. Nutrients.

[55] Rastogi A, Bhansali A, Khare N, Suri V, Yaddanapudi N, Sachdeva N, et al. Short term, high-dose vitamin D supplementation for COVID-19 disease: a randomised, placebo-controlled, study (SHADE study). Postgrad Med J. 2020;139065. 10.1136/postgradmedj-2020-139065 33184146

[56] Murai IH, Fernandes AL, Sales LP, Pinto AJ, Goessler KF, Duran CSC, Silva CBR, Franco AS, Macedo MB, Dalmolin HHH, Baggio J, Balbi GGM, Reis BZ, Antonangelo L, Caparbo VF, Gualano B, Pereira RMR (2021). Effect of a single high dose of vitamin D3 on hospital length of stay in patients with moderate to severe COVID-19: a randomized clinical trial. JAMA..

